# Discovery of superconductivity in Nb_4_SiSb_2_ with a V_4_SiSb_2_-type structure and implications of interstitial doping on its physical properties†

**DOI:** 10.1039/d2tc01510b

**Published:** 2022-07-29

**Authors:** Manuele D. Balestra, Omargeldi Atanov, Robin Lefèvre, Olivier Blacque, Yat Hei Ng, Rolf Lortz, Fabian O. von Rohr

**Affiliations:** Department of Quantum Matter Physics, University of Geneva CH-1211 Geneva Switzerland fabian.vonrohr@unige.ch; Department of Chemistry, University of Zürich CH-8057 Zürich Switzerland; Department of Physics, The Hong Kong University of Science and Technology Clear Water Bay Kowloon Hong Kong

## Abstract

We report on the discovery, structural analysis, and the physical properties of Nb_4_SiSb_2_ – a *hitherto* unknown compound crystallizing in the V_4_SiSb_2_-type structure with the tetragonal space group *I*4/*mcm* and unit cell parameters *a* = 10.3638(2) Å and *c* = 4.9151(2) Å. We find Nb_4_SiSb_2_ to be a metal undergoing a transition to a superconducting state at a critical temperature of *T*_c_ ≈ 1.6 K. The bulk nature of the superconductivity in this material is confirmed by the observation of a well defined discontinuity in specific heat with a normalized specific heat jump of Δ*C*(*T*_c_)/*γT*_c_ = 1.33 mJ mol^−1^ K^−2^. We find that for Nb_4_SiSb_2_, the unoccupied sites on the 4b Wyckoff position can be partially occupied with Cu, Pd, or Pt. Low-temperature resistivity measurements show transitions to superconductivity for all three compounds at *T*_c_ ≈ 1.2 K for Nb_4_Cu_0.2_SiSb_2_, and *T*_c_ ≈ 0.8 K for Nb_4_Pd_0.2_SiSb_2_ as well as for Nb_4_Pt_0.14_SiSb_2_. The addition of electron-donor atoms into these void positions, henceforth, lowers the superconducting transition temperature in comparison to the parent compound.

## Abstract 2

We report on the discovery of Nb_4_SiSb_2_. This compound is a new superconductor with a *T*_c_ of 1.6 K. The channel void positions of this phase can be partly filled with Cu, Pd, or Pt, which leads to a lowering of the transition temperature.

## Graphical abstract

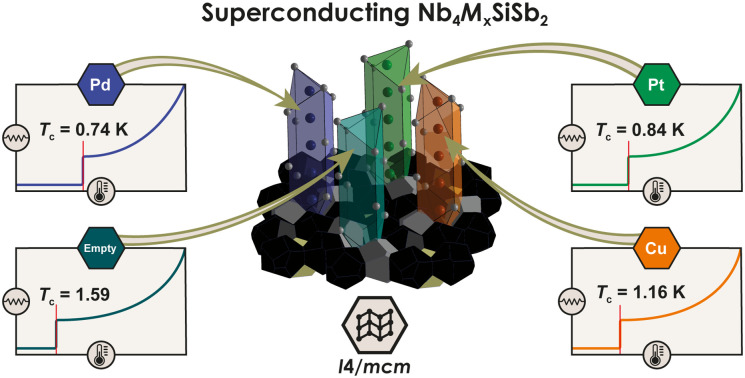

## 1 Introduction

A promising approach for the discovery of new superconducting materials is based on the substitution or incorporation of elements into existing structures with crystallographic void positions. Substituting or incorporating atoms into a structure allows for the precise chemical modification of the density of electronic states at the Fermi-level. This may induce superconductivity or tune superconducting properties.^1–5^ Recent examples of this include the increase in the superconducting transition temperature of Nb_5_Ge_3_ – in the tetragonal Cr_5_B_3_ type-structure – from *T*_c_ ≈ 0.7 K to 15.3 K by the incorporation of carbon atoms into void positions,^6^ or the stabilization of η-carbide superconductors in a filled Ti_2_Ni-type structure, with remarkably high upper critical fields.^4,7^

The results presented in this paper refer to structures, crystallizing in a defect variant of the W_5_Si_3_-type structure, commonly known as the V_4_SiSb_2_ structure. The W_5_Si_3_ structure itself exhibits the tetragonal space group *I*4/*mcm*^8^ and is a bulk superconductor with a critical temperature of *T*_c_ = 2.7 K.^9^ Other compounds crystallizing in the same structure-type and exhibiting superconducting properties are Nb_5_Si_3_ with a critical temperature of *T*_c_ = 0.7 K,^10^ and the ternary W_5_Si_3_-type compounds Nb_5_Sn_2_Ga, Ta_5_SnGa_2_, and Zr_5_Sb_2.36_Ru_0.36_ with critical temperatures of *T*_c_ ≈ 1.8 K, 1.8 K, and 5 K, respectively.^11–13^ In the V_4_SiSb_2_ structure, the 4b Wyckoff position of the W_5_Si_3_ structure is unoccupied, forming void channels along the *c*-direction. These channels are filled by Sb centred, essentially unhybridized 5p orbitals forming a 2D net stacking along the *c*-direction leading to “electron-filled” voids.^14^ The prospect of intercalating these voids with electrophilic species has been theoretically proposed by Rytz *et al.*^14^

To date, only six compounds have been reported to crystallize in this structure-type, namely V_4_SiSb_2_ and the compound series of Ti_4_*T*Bi_2_ with (*T* = Cr, Mn, Fe, Co, Ni). All of these compounds are known to be non-magnetic metals.^15,16^ Furthermore, 5 pseudo-quaternary antimonides with the general formula Nb_4_Pd_0.5_*Z*Sb_2_ with Z = Cr, Fe, Co, Ni, Si have been reported.^17^ These compounds contain three transition metals in an ordered arrangement; hence they are isostructural to each other and crystallize in substitutional variants of the W_5_Si_3_-type structure, or alternatively, they can be interpreted as V_4_SiSb_2_-type compounds with half occupied channels.

Here, we report on the discovery of the compound Nb_4_SiSb_2_, which crystallizes in a V_4_SiSb_2_-type structure with the tetragonal space group *I*4/*mcm*. We show that this material exhibits bulk superconductivity at a critical temperature of *T*_c_ ≈ 1.6 K. Furthermore, we find that the 4b Wyckoff void position can be partially occupied by the transition metals Cu, Pd or Pt, leading to the compounds Nb_4_Cu_0.2_SiSb_2_, Nb_4_Pd_0.2_SiSb_2_, and Nb_4_Pt_0.14_SiSb_2_. All three compounds are bulk superconductors with critical temperatures of *T*_c_ ≈ 1.2 K, 0.8 K, and 0.8 K, respectively.

## 2 Experimental

### Synthesis

Polycrystalline samples of all compounds were obtained by solid state reaction of the pressed elemental powders at high temperatures. These were synthesized using pure elements as received and stored in air of niobium (powder, 99.99%, *Alfa Aesar*), silicon (pieces, 99.95%, *Alfa Aesar*), antimony (shots, 99.999%, *Alfa Aesar*), copper (powder, 99.7%, *Merck*), palladium (powder, 99.999%, *Acros Organics*) and platinum (powder, 99.999%, *Acros Organics*). The elements were thoroughly mixed and ground in their stoichiometric ratios, then pressed into pellets, and subsequently sealed in quartz ampoules under 400 mbar of Ar. The quartz ampoules were heated to *T* = 1100 °C with a heating rate of 180 °C h^−1^, and annealed at this temperature for 7 days.

### Diffraction

Single crystal X-ray diffraction (SXRD) data were collected at *T* = 160(1) K on a *Rigaku* XtaLAB Synergy, Dualflex, Pilatus 200K diffractometer using a monochromatic X-ray source (Cu *K*_*α*_1__ radiation: *λ* = 1.54184 Å) from a micro-focus sealed X-ray tube and cooled using an *Oxford* liquid–nitrogen Cryostream device. The selected suitable single crystals were mounted using polybutene oil. Pre-experiment, data collection, data reduction and analytical absorption correction^18^ were performed with the program suite CrysAlisPro. Using Olex2,^19^ the structure was solved with the SHELXT^20^ small molecule structure solution program and refined with the SHELXL2018/3 program package^21^ by full-matrix least-squares minimization on F2. PLATON^22^ was used to check the result of the X-ray analysis. CCDC 2166026 (for Nb_4_Cu_0.2_SiSb_2_), 2166027 (for Nb_4_Pd_0.2_SiSb_2_), 2166028 (for Nb_4_Pt_0.14_SiSb_2_) and 2166029 (for Nb_4_SiSb_2_).†

Powder X-ray diffraction (PXRD) measurements were performed on a *Rigaku* SmartLab diffractometer using a Cu X-ray source (*K*_*α*_1__ = 1.540600 Å, *K*_*α*_2__ = 1.544430 Å) with Cu*K*_*β*_ filter and collected using a 2*θ* range of 5–100°. The machine is equipped with a 3 kW sealed X-ray tube, CBO optics and a D/teX Ultra 250 silicon strip detector. Data was recorded using the *SmartLab* Studio II software. *Rietveld* refinements were performed using the *FULLPROF* software package^23^ and fitting of the diffracted data was done using the *Thompson-Cox-Hastings* pseudo-*Voigt* function with asymmetry correction.^24^

### Physical properties

Physical property measurements were carried out on sintered, flat pellets. Temperature-dependent resistivity measurements were performed with a Quantum Design Physical Property Measurement System (PPMS) using a He-3 insert for temperature measurements down to 500 mK. A four-point resistivity measurement method, using silver wires (50 μm diameter) was employed.

Specific heat measurements were performed from 300 mK to 2 K in a He-3 15 T magnet cryostat with a custom-developed modulated-temperature AC calorimetry technique using an SR830 digital lock-in amplifier, and from 2–10 K with a long relaxation technique in a He-4 cryostat. For the latter, each relaxation provides about 1000 data points over a temperature interval of 30–40% of the base temperature, which has been varied between 2 K and 10 K. The relaxation technique provides a high precision up to 1% while the AC technique is less accurate but provides high resolutions of Δ*C*/*C* of 10^−5^ at a high density of data points.^25^ Temperature-dependent magnetization measurements were performed using a Quantum Design Magnetic Properties Measurement System (MPMSXL) equipped with a reciprocating sample option (RSO) and a 7 T magnet.

## 3 Results and discussion

### 3.1 Crystal Structure of Nb_4_SiSb_2_

**Fig. 1:**
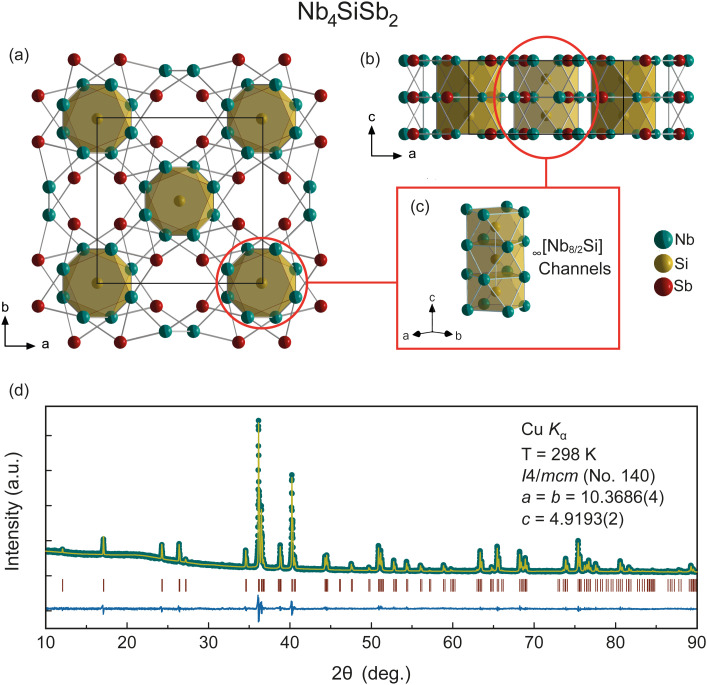
Schematic representation of crystal structure obtained from SXRD refinement of Nb_4_SiSb_2_ along (a) the *c*-direction and (b) the *b*-direction. (c) Si–Si chains along the *c*-direction. (d) PXRD pattern of the polycrystalline sample with the respective *Rietveld* refinement. Green dots: data points, yellow line: calculated peaks, vertical dark red lines: Bragg peak positions, and the blue pattern on the bottom is the difference plot.

In Fig. 1a and b, we present the crystal structure and the unit cell of the single-crystal refinement of Nb_4_SiSb_2_, shown along the *c*-direction and along the *b*-direction, respectively. The structure of Nb_4_SiSb_2_ was determined by means of single crystal X-Ray diffraction (SXRD) at 160 K and the elemental composition was confirmed using EDX analysis at ambient temperature (ESI†).

We find Nb_4_SiSb_2_ to crystallize in the tetragonal space group *I*4/*mcm* with the lattice parameters *a* = *b* = 10.3638(2) Å and *c* = 4.9151(2) Å with the corresponding calculated cell volume of *V* = 527.92(3) Å^3^. Hence, it is found to adopt the same centrosymmetric structure type that was previously reported for V_4_SiSb_2_.^15,16^ The crystallographic data and the details of the structure refinement are summarised in Table 1. All crystallographic positions as well as the anisotropic displacement parameters are presented in Table 2.

**Table 1:** Details of the SXRD measurements and structural refinements for Nb_4_SiSb_2_, Nb_4_Cu_0.2_SiSb_2_, Nb_4_Pd_0.2_SiSb_2_ and Nb_4_Pt_0.14_SiSb_2_

Parameters	Nb_4_SiSb_2_	Nb_4_Cu_0.2_SiSb_2_	Nb_4_Pd_0.2_SiSb_2_	Nb_4_Pt_0.14_SiSb_2_
Crystal system	Tetragonal	Tetragonal	Tetragonal	Tetragonal
Structure-type	V_4_SiSb_2_	W_5_Si_3_(defect)	W_5_Si_3_(defect)	W_5_Si_3_(defect)
Space group	*I*4/*mcm* (No. 140)	*I*4/*mcm* (No. 140)	*I*4/*mcm* (No. 140)	*I*4/*mcm* (No. 140)
Absorption correction method	Analytical	Analytical	Spherical	Analytical
Temperature [K]	160(1)	160(1)	160(1)	160(1)
Lattice parameters [Å]	*a* = 10.3638(2)	*a* = 10.3954(2)	*a* = 10.3991(2)	*a* = 10.3803(2)
	*c* = 4.9151(2)	*c* = 4.9233(2)	*c* = 4.93619(16)	*c* = 4.9348(2)
Cell volume [Å^3^]	527.92(3)	532.03(3)	533.81(3)	531.73(3)
Formula unit/cell	4	4	4	4
*ρ*_*calcd*_ [g cm^−3^]	8.093	8.189	8.268	8.376
*μ* [mm^−1^]	149.393	532.03(3)	153.021	155.001
Crystal size [mm]	0.018 × 0.016 × 0.013	0.005 × 0.003 × 0.002	0.01 × 0.01 × 0.01	0.015 × 0.015 × 0.01
*F*(000)	1120.0	1143.0	1157.0	1164.0
Radiation type	Cu *K*_*α*_ (*λ* = 1.54184)	Cu *K*_*α*_ (*λ* = 1.54184)	Cu *K*_*α*_ (*λ* = 1.54184)	Cu *K*_*α*_ (*λ* = 1.54184)
2*Θ* range [°]	12.078 to 146.58	12.04 to 148.58	12.036 to 147.576	12.058 to 147.716
Index range	*h*[−9,12]	*h*[−11,9]	*h*[−11,12]	*h*[−12,12]
	*k*[−12,12]	*k*[−12,12]	*k*[−12,12]	*k*[−12,12]
	*l*[−5,6]	*l*[−6,6]	*l*[−6,5]	*l*[−6,5]
Observed reflections	1466	838	2368	2381
Independent reflections (2*σ*)	165	166	166	167
*R*_*int*_	0.0278	0.0385	0.0312	0.0298
*R*_*σ*_	0.0127	0.0314	0.0107	0.0117
Refined parameters	14	16	16	17
GOF	1.363	1.142	1.252	1.240
*R*_1_ (all data) (%)	1.69	3.33	1.64	1.60
w*R*_1_ (≥2*σ*) (%)	1.69	2.96	1.62	1.57
w*R*_2_ (all data) (%)	4.32	7.53	3.71	3.67
w*R*_2_ (≥2*σ*) (%)	4.33	7.37	3.71	3.66
Max/min residual electron density [e Å^−3^]	1.41/−0.94	1.12/−1.52	0.97/−0.98	1.13/−0.85

**Table 2:** Atomic coordinates, occupancy, isotropic and anisotropic displacement parameters of the SXRD refinements at 160 K under atmospheric pressure for the compounds Nb_4_SiSb_2_, Nb_4_Cu_0.2_SiSb_2_, Nb_4_Pd_0.2_SiSb_2_ and Nb_4_Pt_0.14_SiSb_2_ (Space Group *I*4/*mcm*, No. 140)

Atom	Wyckoff symbol	*x*	*y*	*z*	*U*(eq) [Å^2^]	*U*_11_/*U*_22_	*U*_33_	*U*_12_	Occ.
Nb_4_SiSb_2_									
Nb	16k	0.29305(6)	0.58530(6)	1/2	0.0111(3)	10.6(4)/10.9(4)	11.8(4)	0.2(2)	4.00
Si	4a	1/2	1/2	3/4	0.0122(3)	10.1(11)/10.1(11)	16(2)	0	1.00
Sb	8h	0.14037(5)	0.35963(5)	1/2	0.0119(9)	11.8(3)/11.8(3)	13.0(5)	−1.5(3)	2.00

Nb_4_Cu_0.2_SiSb_2_									
Nb	16k	0.29297(9)	0.41603(9)	1/2	0.0080(4)	6.2(6)/6.4(6)	11.4(6)	0.3(4)	4.00
Cu	4b	0	1/2	3/2	0.021(7)	24(8)/24(8)	16(12)	0	0.199(16)
Si	4a	1/2	1/2	3/2	0.0063(12)	3.2(17)/3.2(17)	12(3)	0	1.00
Sb	8h	0.14385(8)	0.35615(2)	1/2	0.0110(4)	8.8(5)/8.8(5)	15.3(7)	2.3(4)	2.00

Nb_4_Pd_0.2_SiSb_2_									
Nb	16k	0.29305(4)	0.58369(4)	1/2	0.0125(2)	12.1(3)/12.8(3)	12.7(3)	−0.01(17)	4.00
Pd	4b	0	1/2	1/4	0.0147(15)	13.6(16)/13.6(16)	17(2)	0	0.199(5)
Si	4a	1/2	1/2	3/2	0.0131(6)	13.1(8)/13.1(8)	13.0(14)	0	1.00
Sb	8h	0.14470(4)	0.35530(4)	1/2	0.0170(2)	15.8(2)/15.8(2)	19.5(3)	−3.5(2)	2.00

Nb_4_Pt_0.14_SiSb_2_									
Nb	16k	0.58429(5)	0.29284(5)	1/2	0.0063(2)	6.3(3)/6.2(3)	6.5(4)	−0.08(18)	4.00
Pt	4b	1/2	0	3/2	0.0122(5)	12.5(17)/12.5(17)	12(3)	0	0.140(3)
Si	4a	1/2	1/2	3/2	0.0020(6)	2.7(8)/2.7(8)	0.7(16)	0	1.00
Sb	8h	0.35697(4)	0.14303(4)	1/2	0.0101(3)	9.0(3)/9.0(3)	12.5(4)	−3.1(2)	2.00

In the structure of Nb_4_SiSb_2_ each atom occupies one atomic site: the niobium atoms are located at the 16k Wyckoff position, silicon occupies the 4a and antimony the 8h Wyckoff positions. Silicon forms thereby columns which can be interpreted as _∞_[Nb_8/2_Si] chains along the *c*-direction as shown in Fig. 1(c). The Si–Si bonding distance in Nb_4_SiSb_2_ within the columns is 2.4576(1) Å, which is in good agreement with the ones found in V_4_SiSb_2_^15^ and comparable to Si–Si bond distances in similar structures.^17,26^ Each Si atom is surrounded by eight Nb atoms with a distance of 2.6252(6) Å forming antiprisms with the surrounding neighbour atoms. Nb has a coordination number (CN) of 13 consisting of six Nb neighbours located in the _∞_[Nb_8/2_Si] column, one Nb in the adjacent _∞_[Nb_8/2_Si] column, two Si, and four Sb neighbours located in between the two columns. The Nb–Nb distances range from 3.0275(8) to 3.2807(9) Å. These distances, together with the relatively short intercolumn distance between two Nb atoms of 3.0449(13) Å are in good agreement with distances found in comparable structures.^27,28^ Also, the Nb-Sb distance ranging from 2.8238(7) Å to 2.9781(4) Å is in good agreement with the distances found in the related compounds, such as *e.g.* in Nb_5_Sb_4_.^28^ Each Sb has eight Nb neighbours and therefore a CN of 8. Another feature of this structure are the voids at the 4b Wyckoff position. These void positions are surrounded by four Sb atoms. These form void channels along the *c*-direction. If these void positions were fully occupied, then the V_4_SiSb_2_ structure would be equivalent to the W_5_Si_3_ structure.^15^

The validity of the structural model, the phase purity, and the homogeneity of the sample were confirmed by means of PXRD at ambient temperature and SXRD at 160 K. The reliability factors of the SXRD refinement can be found in the ESI.† In Fig. 3(d) the PXRD pattern of the polycrystalline sample is shown, with its respective *Rietveld* refinement. We find the lattice parameters of *a* = *b* = 10.3686(4) Å, and *c* = 4.9193(2) Å, as well as a calculated cell volume of *V* = 528.86(3) Å^3^. Hence, the SXRD and PXRD refinements and structural solutions are in excellent agreement with each other (ESI†).

### 3.2 Superconducting properties of Nb_4_SiSb_2_

**Fig. 2:**
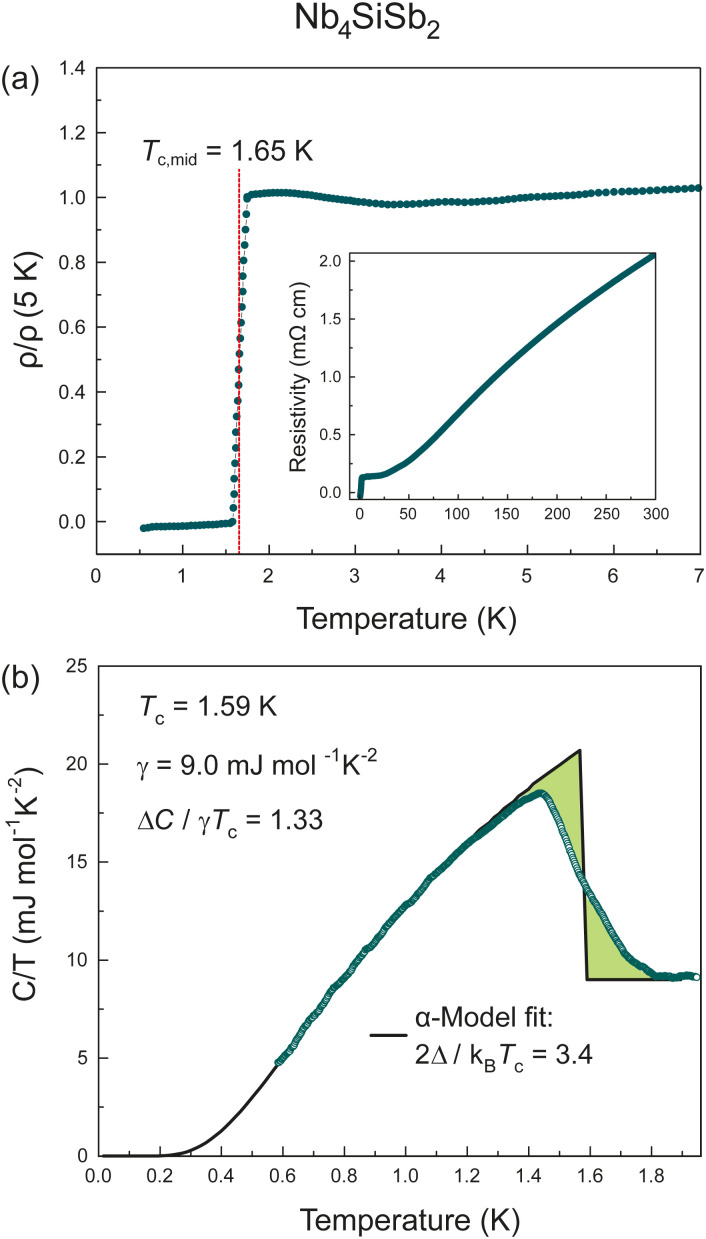
(a) Normalized, low-temperature resistivity of Nb_4_SiSb_2_ in a temperature range between *T* = 500 mK and 5 K measured in zero field *μ*_0_*H* = 0 T. Inset: Temperature-dependant resistivity of Nb_4_SiSb_2_ in zero field *μ*_0_*H* = 0 T as *ρ*(*T*) between *T* = 500 mK and 300 K (b) Specific heat capacity of Nb_4_SiSb_2_ in a temperature range between *T* = 580 mK and 2 K. The black line corresponds to a fit using the α-model.

In Fig. 2(a), we show the temperature-dependant resistivity of Nb_4_SiSb_2_ in zero field *μ*_0_*H* = 0*T* as *ρ*(*T*) between *T* = 300 K and 500 mK (inset) and in the vicinity of the superconducting transition. A sharp drop in the resistivity is observed at low temperature, corresponding to a transition to a superconducting state. The transition midpoint of *T*_c,mid_ ≈ 1.65 K and reaches a state of zero resistance at *T*_zero_ ≈ 1.56 K. The transition is comparably sharp with a transition width of Δ*T* = 0.18 K in the resistivity. The residual resistivity *ρ*(1.8 K) = 0.14 mΩ cm at 1.8 K and the room temperature resistivity value of *ρ*(300 K) = 2.06 mΩ cm, result in a residual resistivity ratio (RRR) here defined as RRR = *ρ*(300 K)/*ρ*(1.8 K) = 14.96. This RRR value corresponds to the value of a good metal.

The bulk nature of the superconductivity in Nb_4_SiSb_2_ is confirmed by low-temperature specific-heat measurements. Temperature-dependent specific-heat measurements are of particular importance to prove the bulk nature of a superconductor.^29,30^

In Fig. 2(b), we present the temperature-dependent specific heat *C*(*T*)/*T* of Nb_4_SiSb_2_ in a temperature range between *T* = 600 mK and 2 K. We find a clearly pronounced discontinuity in the specific heat, resulting from the superconducting transition. The data was fitted using the α-model.^31,32^ Thereby, an entropy conserving construction was used to determine the critical temperature, *T*_c_ ≈ 1.6 K. This value is in good agreement with the critical temperature from the resistivity measurement. From the α-model fit, we obtained *α* = 1.7 and the Sommerfeld constant of *γ* = 9.00 mJ mol^−1^ K^−2^. We find a ratio for the normalized specific-heat jump of Δ*C*/*γT*_c_ = 1.33 mJ mol^−1^ K^−2^, which confirms the bulk nature of the superconductivity, as this value is close to the weak-coupling BCS ratio of 1.43. This corresponds to a value of the superconducting gap of 2*Δ*(0) = 3.4*k*_B_*T*_c_.

Under the assumption of a degenerate electron gas of non-interacting particles, the electronic contribution to the heat capacity in a solid at low temperatures is proportional to the density of states at the *Fermi* level *D*(*E*_F_) and linear in *T*. With the previously determined value of *γ* = 9.00 mJ mol^−1^ K^−2^, the density of states at the *Fermi* level can be calculated as described by F. Heiniger *et al.*^33^ according to1
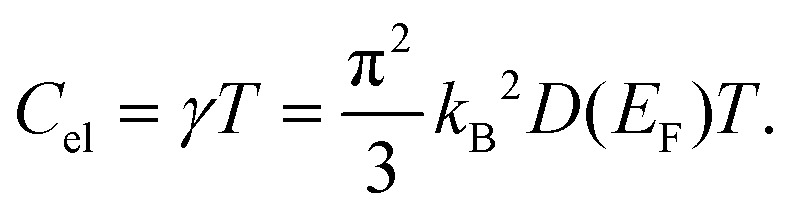


We obtain for Nb_4_SiSb_2_ a density of states at the *Fermi* level of *D*(*E*_*F*_) = 3.8 states eV^−1^.

Magnetic susceptibility measurements of Nb_4_SiSb_2_ were conducted in the normal-state, *i.e.* in a temperature range between *T* = 10 K to 300 K, in an external field of *μ*_0_*H* = 1 T. The observed temperature-independent positive magnetic moment corresponds to a *Pauli*-paramagnet (see ESI†). A summary of all obtained physical parameters can be found in Table 3.

**Table 3:** Summary of the physical parameters for Nb_4_SiSb_2_, Nb_4_Cu_0.2_SiSb_2_, Nb_4_Pd_0.2_SiSb_2_ and Nb_4_Pt_0.14_SiSb_2_

Parameter	Units	Nb_4_SiSb_2_	Nb_4_Cu_0.2_SiSb_2_	Nb_4_Pd_0.2_SiSb_2_	Nb_4_Pt_0.14_SiSb_2_
*T*_c,resistivity_	K	1.65	1.16	0.76	0.84
*T*_c,specificheat_	K	1.59	—	—	—
RRR	—	14.96	4.54	1.56	1.70
*ρ*(300)	mJ Ω cm	2.06	0.70	8.46	2.49
*ρ*_0_	mJ Ω cm	0.13	0.15	5.43	1.46
Type of magnetism	—	*Pauli*-paramagnetic	*Pauli*-paramagnetic	*Pauli*-paramagnetic	*Pauli*-paramagnetic
*γ*	mJ mol^−1^ K^−2^	9.00	7.5	—	
Δ*C*/*T*_c_*γ*	—	1.33	1.2	—	
2*Δ*(0)	meV	0.47	12	—	
*D*(*E*_*F*_)	States eV^−1^ per f.u.	3.82	3.18	—	

### 3.3 Crystal Structures of Nb_4_Cu_0.2_SiSb_2_, Nb_4_Pd_0.2_SiSb_2_, and Nb_4_Pt_0.14_SiSb_2_

**Fig. 3:**
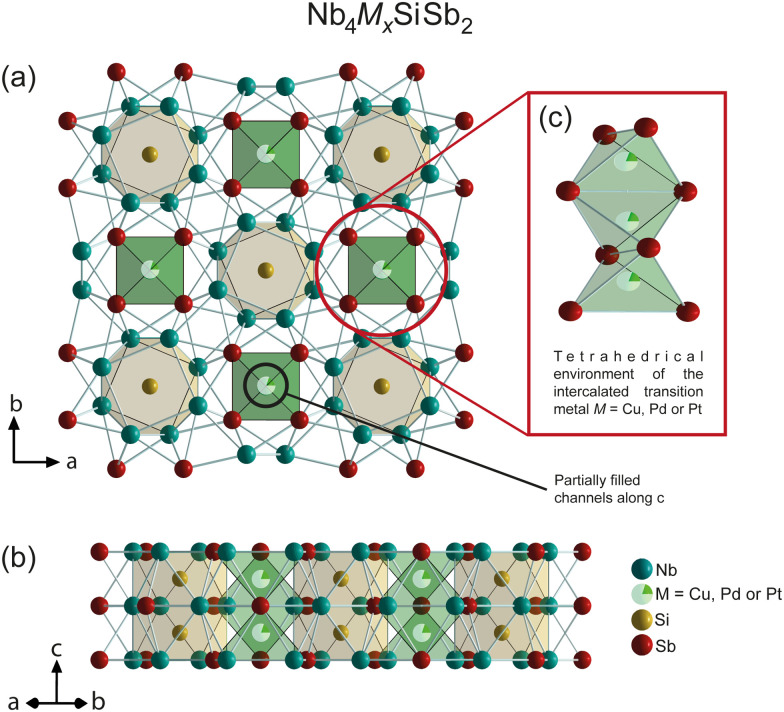
Schematic representation of the structures of Nb_4_M_*x*_SiSb_2_ from (a) the *c*-direction and (b) a high symmetry-direction. (c) illustrates the intercalated transition metal M (Cu, Pd, or Pt) in its environment within the _∞_[Sb_4/2_M_*x*_] channels. The crystal structures were obtained from SXRD refinements.

We have synthesized the three compounds Nb_4_Cu_0.2_SiSb_2_, Nb_4_Pd_0.2_SiSb_2_, and Nb_4_Pt_0.14_SiSb_2_. Here, the void 4b Wyckoff positions in Nb_4_SiSb_2_ are partially filled with a transition metal *M* = Cu, Pd, or Pt, respectively. In Fig. 3, we show a schematic representation of the unit cell along the *c*-direction and a high symmetry-direction of Nb_4_*M*_*x*_SiSb_2_, where *M* = Cu, Pt and Pd with *x* = 0.2, 0.14 and 0.2. The crystal structures of all three compounds were determined using SXRD at 160 K and PXRD diffraction at room temperature.

All samples were found to be single phase by means of PXRD measurements and corresponding Rietveld refinements (ESI†). Atomic compositions were confirmed using EDX analysis (ESI†).

All three structures are in good agreement with the previously reported structure for Nb_4_Pd_0.5_*Z*Sb_2_ with *Z* = Cr, Fe, Co, Ni, Si, where it was thought that a half-occupied Pd 4b site was necessary to stabilize these compounds.^17^ In contrary to this previous assumption, we found here that the channels were in our case either unoccupied or filled with 0.2 or 0.14 respectively (in case of Pt), independent of the initially used starting stoichiometry. These results indicate that, with improved synthesis methodologies, the continuous solid solution might be accessible in the future. All information regarding the lattice parameters, crystallographic data, and details of the structure refinements are summarized in Table 1.

### 3.4 Electronic properties of Nb_4_Cu_0.2_SiSb_2_, Nb_4_Pd_0.2_SiSb_2_ and Nb_4_Pt_0.14_SiSb_2_

**Fig. 4:**
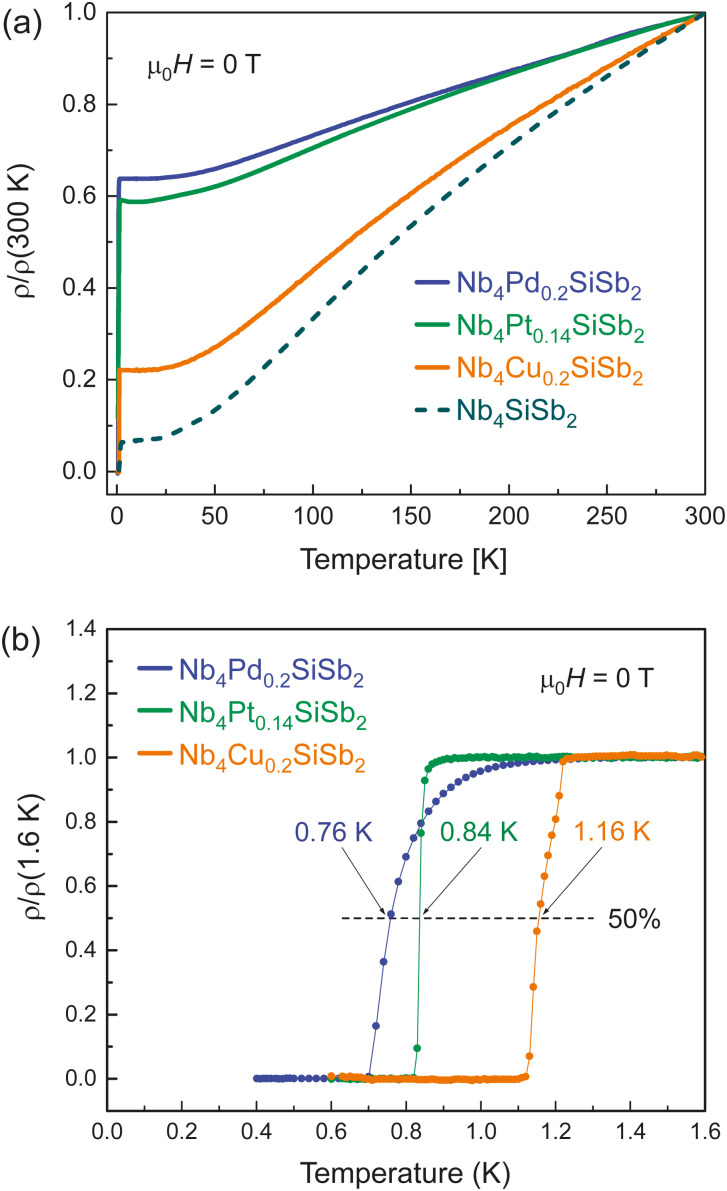
(a) Temperature-dependent resistivity of Nb_4_Cu_0.2_SiSb_2_, Nb_4_Pd_0.2_SiSb_2_ and Nb_4_Pt_0.14_SiSb_2_ (a) over the whole temperature range between *T* = 400 mK and 300 K, and (b) in the vicinity of the superconducting transitions at low temperatures. All measurements were performed in zero field *μ*_0_*H* = 0 T.

In Fig. 4 we present the temperature-dependent resistivity and the normalized low-temperature resistivity *ρ*(*T*)/*ρ*(1.6*K*) in a temperature range between *T* = 400 mK and 1.6 K for Nb_4_Cu_0.2_SiSb_2_, Nb_4_Pd_0.2_SiSb_2_ and Nb_4_Pt_0.14_SiSb_2_, measured in zero field *μ*_0_*H* = 0 T.

We find all three compounds to undergo a transition to a superconducting state at low temperatures. The critical temperature midpoints are determined as *T*_c,mid_ ≈ 1.16 K for Nb_4_Cu_0.2_SiSb_2_, *T*_c,mid_ ≈ 0.76 K for Nb_4_Pd_0.2_SiSb_2_ and *T*_c,mid_ ≈ 0.84 K for Nb_4_Pt_0.14_SiSb_2_. All three compounds with atoms in the void position of Nb_4_SiSb_2_ have lower transition temperatures than the parent compound.

For comparison, we have performed specific heat measurements in the normal state of Nb_4_SiSb_2_ and Nb_4_Pt_0.14_SiSb_2_ (shown in the ESI†). For Nb_4_SiSb_2_ we find values for *γ*_*n*_ and *β* of 8.40 mJ mol^−1^ K^−2^ and 0.16 mJ mol^−1^ K^−4^, respectively. The *γ*_*n*_ value of this fit is in good agreement with the more accurate low-temperature value discussed above. For Nb_4_Pt_0.14_SiSb_2_ we find values for *γ*_*n*_ and *β* of 9.10 mJ mol^−1^ K^−2^ and 0.31 mJ mol^−1^ K^−4^, respectively. We note that the values for *γ*_*n*_ differ only slightly, indicating a small change of the electronic properties upon void position filling. We find, however, that *β* changes quite strongly. These findings indicate that the decrease of the superconducting transition temperature is likely caused by a change in the phonons, and the vibrations, respectively.

Nb_4_Pd_0.2_SiSb_2_ has the lowest critical temperature of the doped compounds, as well as the lowest RRR value of RRR = *ρ*(300 K)/*ρ*(1.8 K) = 1.56. Nb_4_Cu_0.2_SiSb_2_ with RRR = 4.54 and Nb_4_Pt_0.14_SiSb_2_ with RRR = 1.70 follow the descending trend observed for the critical temperatures accordingly. These low RRR values correspond to a poor metallic behaviour and are 3 to 24 times smaller than the RRR of the parent compound Nb_4_SiSb_2_. The pronounced effect on the physical properties on void position doping becomes clearly apparent in the large change of the RRR values. The nature of the change is, however, not only affected by the electronic states, but also by the phonons and by impurity state scattering.

## 4 Conclusion

We have reported on the discovery of the centrosymmetric structure compound Nb_4_SiSb_2_. This phase was found to crystallize in the tetragonal V_4_SiSb_2_-type structure. We found Nb_4_SiSb_2_ to undergo a transition to a superconducting state at a critical temperature of *T*_c_ ≈ 1.6 K. The bulk nature of the superconducting transition was evidenced by a clear discontinuity in specific heat, with a normalized specific heat jump of Δ*C*(*T*_c_)/*γT*_c_ = 1.33 mJ mol^−1^ K^−2^, close to the weak-coupling BCS value. Furthermore, we have shown that the unoccupied 4b Wyckoff site in Nb_4_SiSb_2_ can be partially occupied with the transition metals Cu, Pd, or Pt.

These compounds crystallize in a tetragonal variant of the W_5_Si_3_-type structure with partially occupied channels, extending along the *c*-direction. All three compounds were found to be superconductors with transitions temperatures of *T*_c_ ≈ 1.2 K for Nb_4_Cu_0.2_SiSb_2_, *T*_c_ ≈ 0.8 K for Nb_4_Pd_0.2_SiSb_2_ and *T*_c_ ≈ 0.8 K for Nb_4_Pt_0.14_SiSb_2_. We find that the insertion of a host atom into the void positions strongly affects the electronic and superconducting properties of this material.

Hence, our results indicate that this and related compounds might be promising host structures for the discovery of new superconducting materials, as they allow for a controlled manipulation of the electronic and phononic properties by chemical manipulation.

## Conflicts of interest

There are no conflicts to declare.

## Supplementary Material

TC-010-D2TC01510B-s001

TC-010-D2TC01510B-s002
