# Supplementary material for: Discovery of superconductivity in Nb4SiSb2 with a V4SiSb2-type structure and implications of interstitial doping on its physical properties
Source: J Mater Chem C Mater. 2022 Jul 29;10(32):11703–9. doi: 10.1039/d2tc01510b (PMC9386687; doi:10.1039/d2tc01510b)
Supplement: TC-010-D2TC01510B-s001 [file TC-010-D2TC01510B-s001.pdf]

Electronic Supplementary Material (ESI) for Journal of Materials Chemistry C.
This journal is © The Royal Society of Chemistry 2022

Supplementary Information on: Discovery of Superconductivity in Nb_4SiSb_2 with a V_4SiSb_2-Type Structure and Implications of Interstitial Doping on its Physical Properties

Manuele D. Balestra,[1] Omargeldi Atanov,[2] Robin Lefèvre,[3] Olivier Blacque,[3] Yat H. Ng,[2] Rolf Lortz,[2] and Fabian O. von Rohr[1,*]

[1] *Department of Quantum Matter Physics, University of Geneva, CH-1211 Geneva, Switzerland*
[2] *Department of Physics, The Hong Kong University of Science and Technology, Clear Water Bay Kowloon, Hong Kong*
[3] *Department of Chemistry, University of Zurich, CH-8057 Zurich, Switzerland*

(Dated: July 8, 2022)

I. STRUCTURE REFINEMENTS

A. Powder X-Ray Diffraction Refinements

In Figure 1, we show the results of the PXRD *Rietveld* refinements for $Nb_4Cu_{0.2}SiSb_2$, $Nb_4Pd_{0.2}SiSb_2$, and $Nb_4Pt_{0.14}SiSb_2$. Further structural details, general parameters and reliability factors can be found in Table I. Furthermore, Table II includes all the SCXRD refinement data.

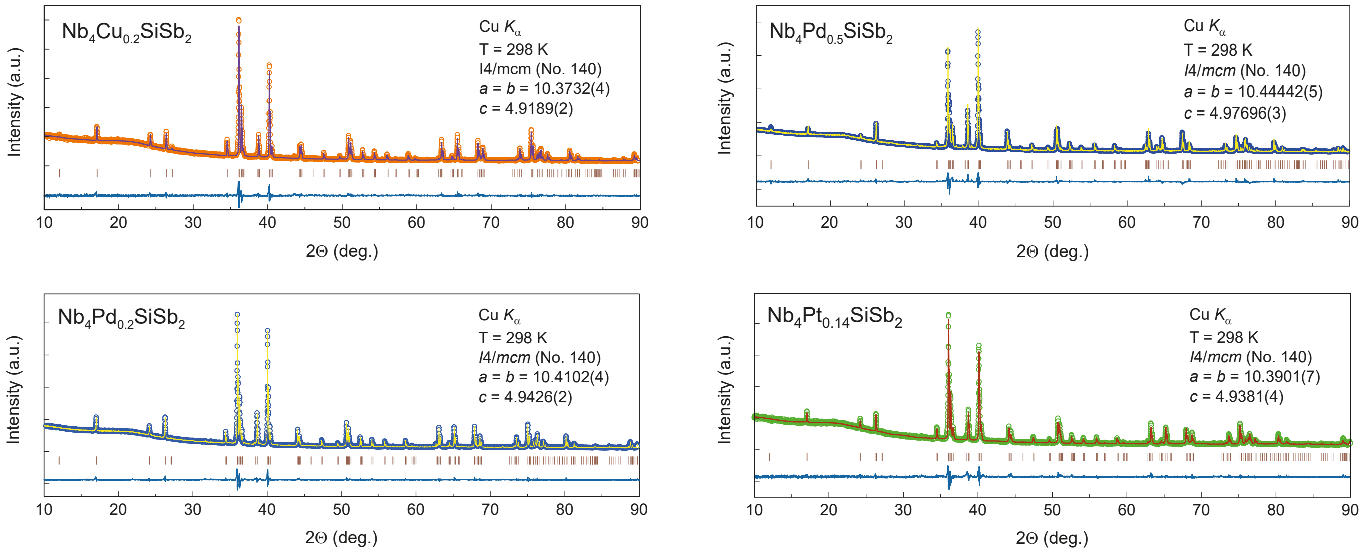

FIG. 1. Results from the *Rietveld* refinements of $Nb_4Cu_{0.2}SiSb_2$, $Nb_4Pd_{0.2}SiSb_2$, and $Nb_4Pt_{0.14}SiSb_2$. Round data points are the measured data points, the lines are the refinements, vertical dark red lines theoretical Bragg peak positions, blue pattern on the bottom is the difference plot between the theoretical pattern and the observed intensities.

* fabian.vonrohr@uzh.ch

TABLE I. Details on the PXRD structure refinements of the polycrystalline samples for Nb_4SiSb_2, $Nb_4Cu_{0.2}SiSb_2$, $Nb_4Pd_{0.2}SiSb_2$, and $Nb_4Pt_{0.14}SiSb_2$.

General parameters	
Radiation source	Cu-K_α
Wavelengths [Å^2]	1.54060, 1.54443
Temperature [K]	298
2Θ range, No. of points	5–100°, 9493
Step size, counting time	0.010°, 2.0 s
Background function	Linear interpolation
Profile function	*Thompson-Cox-Hastings* pseudo-*Voigt*

Compounds		Nb_4SiSb_2	$Nb_4Cu_{0.2}SiSb_2$	$Nb_4Pd_{0.2}SiSb_2$	$Nb_4Pt_{0.14}SiSb_2$
No. of refined parameters		12	14	13	13
Unit-cell parameters [Å]	a	10.3686(4)	10.3732(4)	10.4102(4)	10.3901(7)
	c	4.9193(2)	4.9189(2)	4.9426(2)	4.9381(4)
Cell volume [A^3]		528.87(4)	529.29(4)	535.64(3)	533.10(7)
Reliability factors	R_F (%)	9.83	13.1	12.1	18.6
	R_B (%)	8.80	10.7	8.22	12.5
	R_P (%)	3.59	3.39	2.54	3.68
	R_{WP} (%)	5.41	5.01	4.23	5.44
	χ^2	2.54	2.10	5.95	2.42
	GoF	1.6	1.4	2.4	1.6

TABLE II. Details of the single-crystal X-ray diffraction measurements and structural refinements for Nb_4SiSb_2, $Nb_4Cu_{0.2}SiSb_2$, $Nb_4Pd_{0.2}SiSb_2$, and $Nb_4Pt_{0.14}SiSb_2$

Parameters	Nb_4SiSb_2	$Nb_4Cu_{0.2}SiSb_2$	$Nb_4Pd_{0.2}SiSb_2$	$Nb_4Pt_{0.14}SiSb_2$
Crystal system	tetragonal	tetragonal	tetragonal	tetragonal
Structure-type	V_4SiSb_2	W_5Si_3(defect)	W_5Si_3(defect)	W_5Si_3(defect)
Space group	$I4/mcm$ (No. 140)	$I4/mcm$ (No. 140)	$I4/mcm$ (No. 140)	$I4$/mcm (No. 140)
Absorption correction method	analytical	analytical	spherical	analytical
Temperature [K]	160(1)	160(1)	160(1)	160(1)
Lattice parameters [Å]	a = 10.3638(2)	a = 10.3954(2)	a = 10.3991(2)	a = 10.3803(2)
	c = 4.9151(2)	c = 4.9233(2)	c = 4.93619(16)	c = 4.9348(2)
Cell volume [Å^3]	527.92(3)	532.03(3)	533.81(3)	531.73(3)
Formula unit/cell	4	4	4	4
ρ_{calcd} [g / cm^3]	8.093	8.189	8.268	8.376
μ [mm^{-1}]	149.393	532.03(3)	153.021	155.001
Crystal size [μm]	18 x 16 x 13	5 x 3 x 2	10 x 10 x 10	15 x 15 x 10
$F(000)$	1120.0	1143.0	1157.0	1164.0
Radiation type	Cu K_α (λ = 1.54184)			
2 Θ range [°]	12.078 to 146.58	12.04 to 148.58	12.036 to 147.576	12.058 to 147.716
Index range	h[-9,12]	h[-11,9]	h[-11,12]	h[-12,12]
	k[-12,12]	k[-12,12]	k[-12,12]	k[-12,12]
	l[-5,6]	l[-6,5]	l[-6,6]	l[-6,5]
Observed reflections	1466	838	2368	2381
Independent reflections	165	166	166	167
R_{int}	0.0278	0.0385	0.0312	0.0298
R_σ	0.0127	0.0314	0.0107	0.0117
Refined parameters	14	16	16	17
GOF	1.363	1.142	1.252	1.240
R_1 (all data)	0.0169	0.0333	0.0164	0.0160
wR_1 ($\geq 2\sigma$)	0.0169	0.0296	0.0162	0.0157
wR_2 (all data)	0.0432	0.0753	0.0371	0.0367
wR_2 ($\geq 2\sigma$)	0.0433	0.0737	0.0371	0.0366
Max/min residual electron density [e Å^{-3}]	1.41/-0.94	1.12/-1.52	0.97/-0.98	1.13/-0.85

II. TEMPERATURE-DEPENDENT SPECIFIC HEAT CAPACITY MEASUREMENTS

Figure 2 shows the temperature-dependent specific heat capacity measurements for $Nb_4Cu_{0.2}SiSb_2$ near the superconducting transition between $T = 500$ mK and 1.6 K. The clearly pronounced discontinuity in the specific heat confirms the bulk nature of the superconducting transition with a transition temperature at $T = 1.17\,K$. The black line shows the fitting curve from the α-model fit resulting in a normalised specific-heat jump of $\Delta C(T_c)/\gamma T_\mathrm{c} = 1.2\,mJ\,mol^{-1}\,K^{-2}$, a superconducting gap of $2\Delta(0) = 3.2\,k_B$ and the *Sommerfeld* parameter $\gamma = 7.5\,mJ\,mol^{-1}\,K^{-1}$.

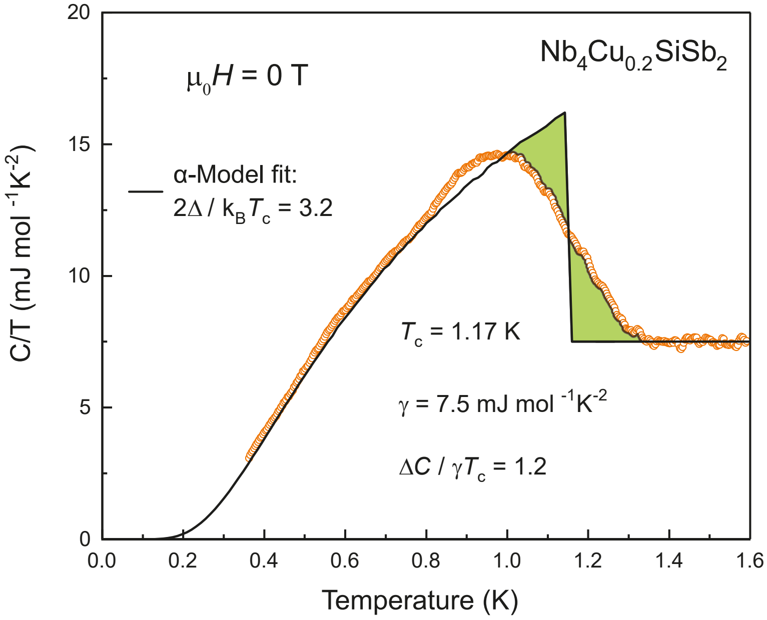

FIG. 2. Temperature-dependent specific heat capacity measurements in a temperature range between 0.5 mK and 1.6 K in zero magnetic field $\mu_0 H = 0\,T$ for $Nb_4Cu_{0.2}SiSb_2$. The black line corresponds to a fit using the α-model.

The normal-state specific heat is analyzed in a standard way according to the expansion

$$C_n(T \to 0) = \gamma_n T + \sum_{n=1}^{3} \beta_{2n+1} T^{2n+1} \tag{1}$$

In which the first term is the electronic contribution, with the Sommerfeld constant $\gamma = \frac{1}{3}\pi^2 k_B^2 (1 + \lambda_{ep}) N(E_F)$, k_B is Boltzmann's constant, γ_{ep} the electron-phonon coupling constant and $N(E_F)$ the electronic density of states at the Fermi level with two spin directions. The second term is the low-temperature expansion of the phonon specific heat, with $\beta_3 = \frac{12}{5} N_{A\nu} k_B n \pi^4 \theta_D^{-3}(0)$, with $N_{A\nu}$ Avogadro's number and $\theta_D(0)$ the initial Debye temperature and n the number of atoms per formula unit. From a fit of the normal-state specific heat above T_c from 2 to 7 K (Fig. 3), we obtain $\gamma_n = 8.4$ mJ K^{-2} mol^{-1}, $\beta_3 = 0.17$ mJ K^{-4} mol^{-1} and $\theta_D(0) = 439.7$ K for Nb_4SiSb_2. For $Nb_4Pt_{1.4}SiSb_2$, for which we also have detailed normal state data, we obtain $\gamma_n = 9.1$ mJ K^{-2} mol^{-1}, $\beta_3 = 0.31$ mJ K^{-4} mol^{-1} and $\theta_D(0) = 355.1$ K. This shows that, while the electronic contribution is hardly affected by the Pt doping, some low-lying phonon modes become noticeably reduced in energy, which likely explains the decrease in T_c for our doped samples.

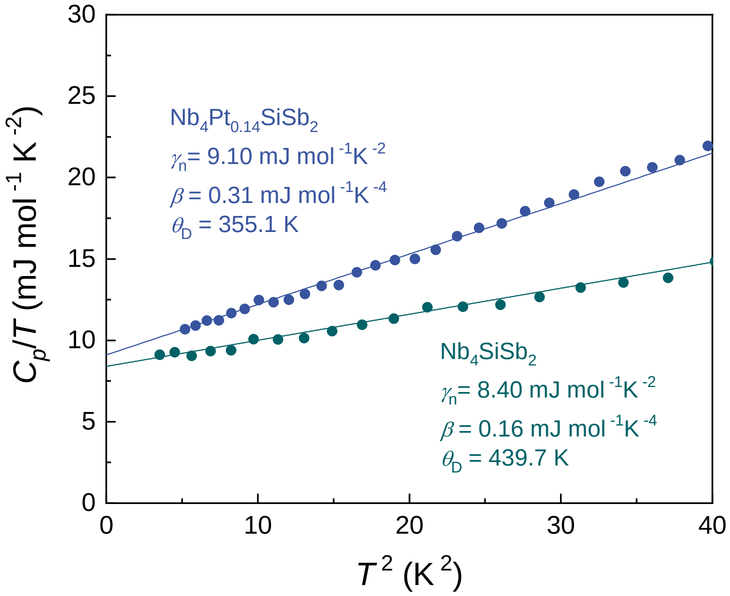

FIG. 3. Normal state specific heat C_p/T vs T^2 for Nb_4SiSb_2 and $Nb_4Pt_{1.4}SiSb_2$ with linear fits according to the low-temperature expansion of the specific heat (see text for details).

III. MAGNETIC SUSCEPTIBILITY MEASUREMENTS

The temperature-dependent magnetization measurements in the normal state of Nb_4SiSb_2, $Nb_4Cu_{0.2}SiSb_2$, $Nb_4Pd_{0.2}SiSb_2$, and $Nb_4Pt_{0.14}SiSb_2$ were performed in a temperature range from 10 to 300 K in a magnetic field $\mu_0 H = 1\,T$. In Figure 4 we show that no temperature-dependence in the magnetization was observed. This indicates *Pauli*-paramagnetism for all compounds.

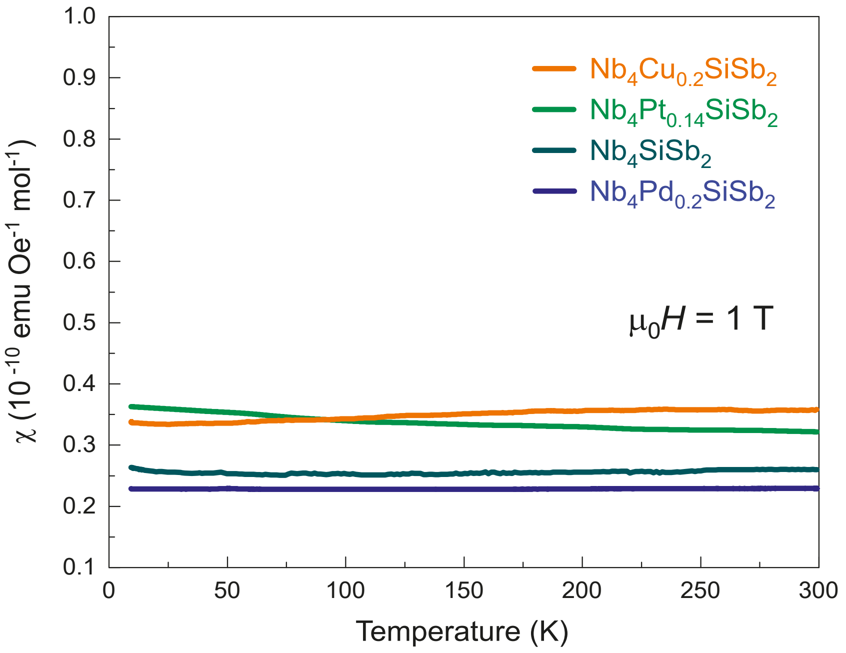

FIG. 4. Plots of the normal state temperature-dependent magnetization measurements in a temperature range between 10 and 300 K in a magnetic field $\mu_0 H = 1\,T$ for Nb_4SiSb_2, $Nb_4Cu_{0.2}SiSb_2$, $Nb_4Pd_{0.2}SiSb_2$, and $Nb_4Pt_{0.14}SiSb_2$. No temperature dependence in magnetization was observed, which indicates all samples being *Pauli*-paramagnetic.

IV. SEM AND EDX ANALYSIS

Scanning electron microscopy (SEM) images were taken with a Zeiss GeminiSEM 450 scanning electron microscope. The elemental composition was determined by energy dispersive X-ray (EDX) analysis on a AZTec Advanced X-MAX80 detector, attached to the Zeiss GeminiSEM 450, using the OXFORD Instruments software AZtecLive. The atomic composition of Nb_4SiSb_2, $Nb_4Cu_{0.2}SiSb_2$, $Nb_4Pd_{0.2}SiSb_2$, and $Nb_4Pt_{0.14}SiSb_2$ was confirmed using EDX analysis. In Figures 5(a) to 8(a), we show the SEM images with the different points which were used for the data collection for the elemental analysis. Further, we show the atomic distribution within the compounds in Figures 5(b) to 8(b) with the lower pictures illustrating the selected energies of the individual elements within the compounds. The results of the EDX analysis are summarized in Table III.

TABLE III. Results on the EDX analysis of Nb_4SiSb_2, $Nb_4Cu_{0.2}SiSb_2$, $Nb_4Pd_{0.2}SiSb_2$ and $Nb_4Pt_{0.14}SiSb_2$.

Nb_4SiSb_2	No. of points for data collection: 12		
Element	Nb	Si	Sb
Atomic ratio	4.04	1.10	2.00
Stand. Dev.	0.02	0.06	0.06

$Nb_4Cu_{0.2}SiSb_2$	No. of points for data collection: 13			
Element	Nb	Cu	Si	Sb
Atomic ratio	3.97	0.14	0.99	2.04
Stand. Dev.	0.04	0.08	0.06	0.05

$Nb_4Pd_{0.2}SiSb_2$	No. of points for data collection: 10			
Element	Nb	Pd	Si	Sb
Atomic ratio	3.91	0.21	1.04	1.99
Stand. Dev.	0.02	0.01	0.04	0.05

$Nb_4Pt_{0.14}SiSb_2$	No. of points for data collection: 13			
Element	Nb	Pt	Si	Sb
Atomic ratio	3.94	0.16	1.06	1.98
Stand. Dev.	0.06	0.04	0.06	0.05

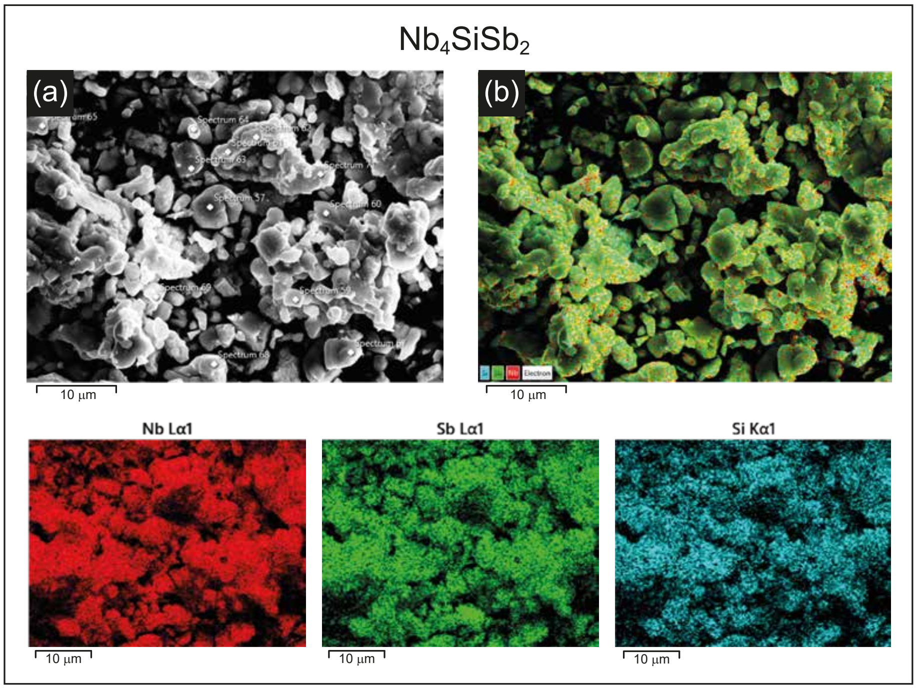

FIG. 5. (a) SEM image of Nb_4SiSb_2 with the 12 points which were used for the EDX analysis. (b) shows a coloured map of the powder sample and the three lower images illustrate the equal distribution of the different elements in the sample.

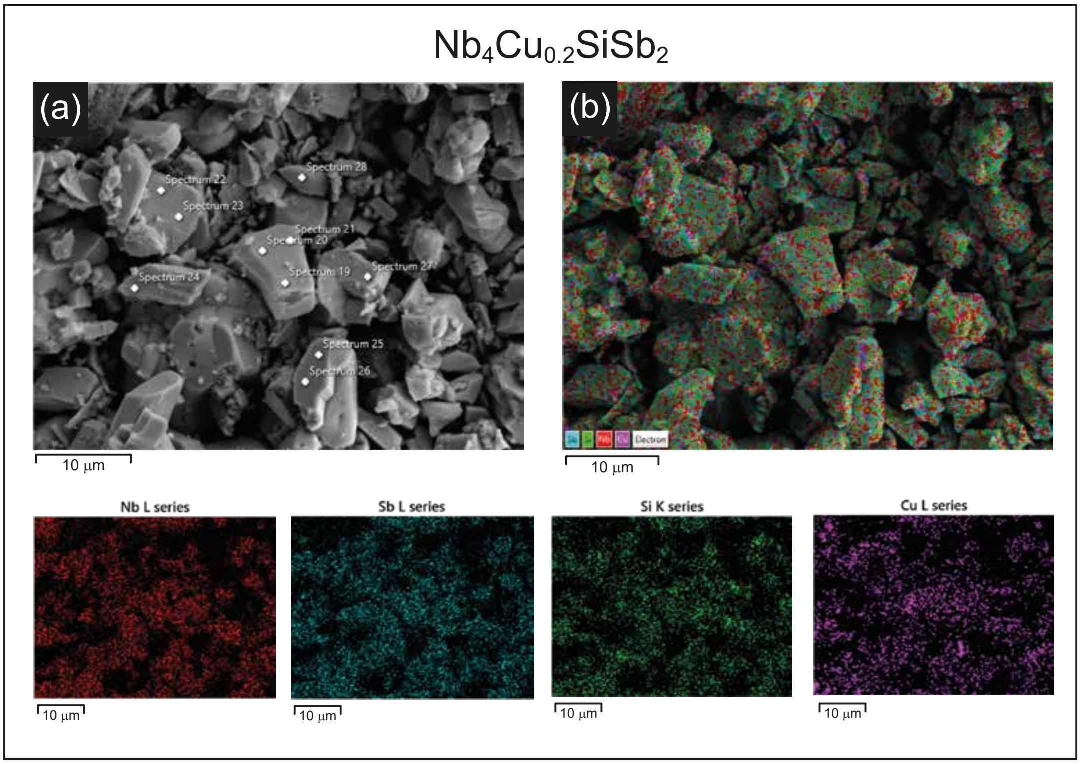

FIG. 6. (a) SEM image of $Nb_4Cu_{0.2}SiSb_2$ with the points which were used for the EDX analysis. (b) shows a coloured map of the powder sample and the four lower images illustrate the equal distribution of the different elements in the sample.

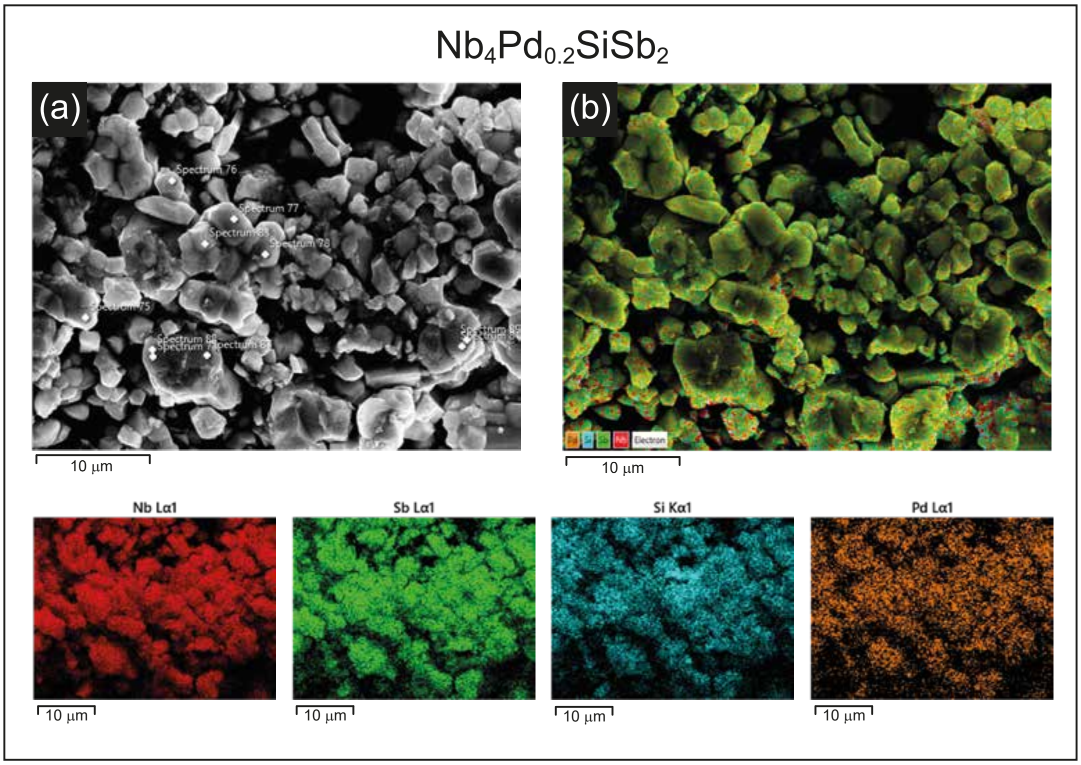

FIG. 7. (a) SEM image of $Nb_4Pd_{0.2}SiSb_2$ with the points which were used for the EDX analysis. (b) shows a coloured map of the powder sample and the four lower images illustrate the equal distribution of the different elements in the sample.

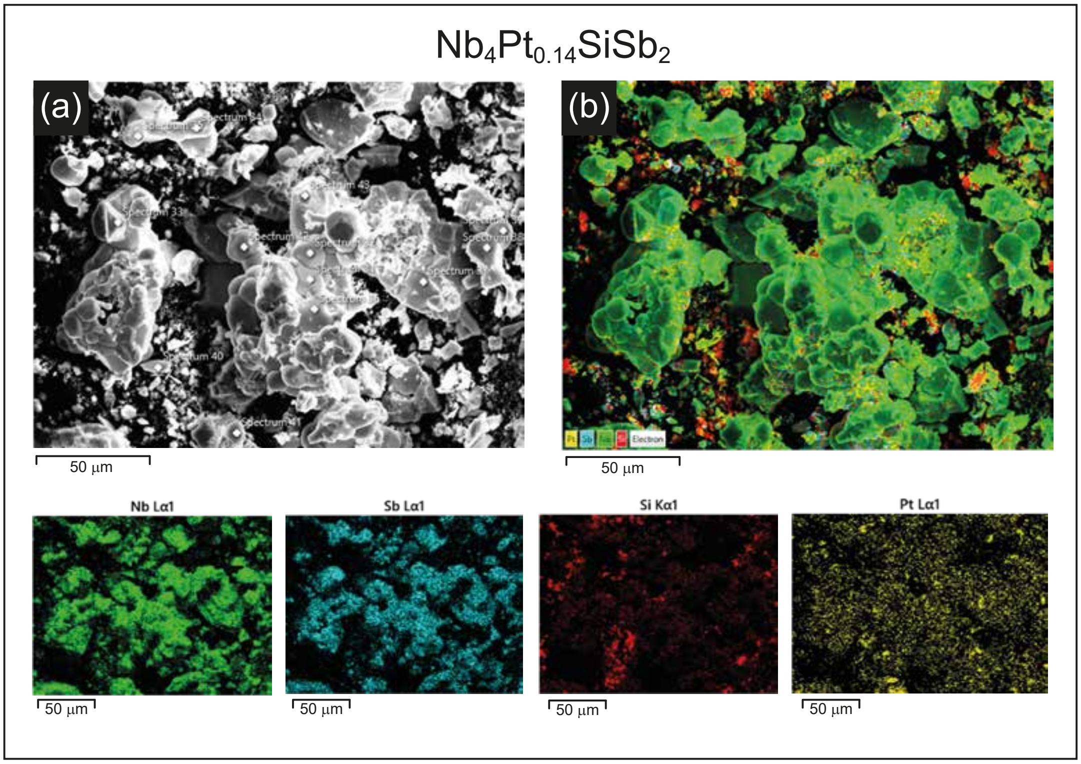

FIG. 8. (a) SEM image of $Nb_4Pt_{0.14}SiSb_2$ with the points which were used for the EDX analysis. (b) shows a coloured map of the powder sample and the lower images illustrate the equal distribution of the different elements in the sample.
